# An assessment of spatio-temporal relationships between nocturnal bird migration traffic rates and diurnal bird stopover density

**DOI:** 10.1186/s40462-015-0066-1

**Published:** 2016-01-07

**Authors:** Kyle G. Horton, W. Gregory Shriver, Jeffrey J. Buler

**Affiliations:** Department of Entomology and Wildlife Ecology, University of Delaware, 531 South College Avenue, Newark, DE 19716 USA; Department of Biology, University of Oklahoma, Norman, OK USA; Oklahoma Biological Survey, University of Oklahoma, Norman, OK USA; Advanced Radar Research Center, University of Oklahoma, Norman, OK USA

**Keywords:** Bird migration, NEXRAD, Quantification, Stopover, Thermal imaging, Weather surveillance radar

## Abstract

**Background:**

Daily magnitudes and fluxes of landbird migration are often measured via nocturnal traffic rates aloft or diurnal densities within terrestrial habitats during stopover. However, these measures are not consistently correlated and at times reveal opposing trends. For this reason we sought to determine how comparison methods (daily magnitude or daily flux), nocturnal monitoring tools (weather surveillance radar, WSR; thermal imaging, TI), and temporal scale (preceding or following diurnal sampling) influenced correlation strength from stopover densities estimated by daily transect counts. We quantified nocturnal traffic rates at two temporal scales; averaged across the entire night and within individual decile periods of the night, and at two spatial scales; within 1 km of airspace surrounding the site via WSR and directly overhead within the narrow beam of a TI.

**Results:**

Overall, the magnitude of daily bird density during stopover was positively related to the magnitude of broad-scale radar traffic rates of migrants on preceding and following nights during both the spring and fall. These relationships were strongest on the following night, and particularly from measures early in the night. Only during the spring on the following nights did we find positive correlations between the daily flux of transect counts and migration traffic rates (both WSR and TI). This indicates that our site likely had a more consistent daily turnover of migrants compared to the fall. The lack of general correlations between seasonal trends or daily flux in fine-scale TI traffic rates and stopover densities across or within nights was unexpected and likely due to poor sampling of traffic rates due to the camera’s narrow beam.

**Conclusions:**

The order (preceding or following day) and metric of comparisons (magnitude or flux), as well as the tool (WSR or TI) used for monitoring nocturnal migration traffic can have dramatic impacts when compared with ground-based estimates of migrant density. WSR provided measures of the magnitude and daily flux in nocturnal migration traffic rates that related to daily stopover counts of migrants during spring and fall. Relationships among migrating bird flux measures are more complex than simple measures of magnitude of migration. Care should be given to address these complexities when comparing data among methods.

## Background

Each year billions of migratory birds make flights to and from their breeding and wintering grounds. Documenting and understanding these flights, especially nocturnal movements, has remained a logistic and technological challenge. Bird density estimates measured during migratory stopover have historically been used to document the passage of nocturnally migrating landbirds [1]. Methods to measure diurnal ground-based avian densities or abundance have included, although are not limited to, the capture of birds using mist-nets and visual surveys (e.g., transect counts, point counts) [1–3]. Whereas these direct capture and visual methods provide detailed species information at migratory stopover sites, and the only means of determining specific-specific age, sex, and physiological condition; tools for measuring traffic rates of migrating birds during nocturnal flight often fail to provide equivalent species information [4]. Techniques for quantifying nocturnal migration include remote sensing tools like low- and high-power radars (e.g., [5, 6]), thermal imaging (e.g., [7, 8]), and acoustic monitoring [9]. Since no single tool yields a complete picture of migration, it is crucial to understand how techniques with fundamentally different sampling methodologies compare.

Positive relationships are frequently found among diurnal metrics (e.g., mist-netting and visual counts; [10]) and among nocturnal metrics (e.g., radar and thermal-imaging [9, 11, 12]), however correlations between diurnal and nocturnal metrics have frequently been inconsistent or non-existent. For example, positive correlations between the magnitude of nocturnal traffic rates detected by low-power X-band radar [10] thermal infrared camera [13], and weather surveillance radar [14] have been linked to diurnal stopover intensity estimated by mist-netting [10, 13, 14] and ground surveys [10, 14]. Yet conversely the magnitude of ceilometer- and mobile radar-based traffic rates were not correlated with mist-net capture rates during the following day during spring migration [11].

Most studies have focused on correlating the magnitudes of traffic rates and stopover incidence among days within a migration period. Traffic rates and stopover incidence vary over the course of a migration period in a cyclical fashion (i.e., are non-stationary). Analyses of non-stationary time-series may be spurious in that they may indicate a relationship between two variables where one does not exist [15]. Calculating differences in a time-series from one period to the next (e.g., daily flux in traffic rates and stopover incidence) is a way to make the data stationary and to obtain meaningful correlations among variables. The two studies that have tested relationships of daily fluxes of traffic and stopover metrics between days have not found positive relationships [16, 17]. Nisbet and Drury [16] did not find a relationship between daily flux in mist-net captures and nocturnal traffic rates from moon watching. Fischer et al. [17] found that within-season daily flux of stopover densities from visual counts at three sites were not related to the magnitude of radar traffic rate (i.e., not a comparable flux of traffic rate) during preceding nights or following nights in spring and fall. Thus, more rigorous comparisons of migration flux on the ground with flux in the air are needed.

In order to develop more consistent and robust estimates of migration traffic rates we need a better understanding of what is measured by each technique and the circumstances (e.g., season, scale, sampling time, etc.) under which different techniques produce correlated measures. Here our main objective was to compare the daily magnitude and flux of two different nocturnal passage metrics with diurnal stopover density estimates based on daily transect counts during both the spring and fall at a single site. Additionally, we compared a single season of fall mist-net captures with ground surveys to investigate commensurate diurnal techniques. We used nocturnal traffic estimates measured at a fine spatial scale by a thermal infrared camera (TI) and at a coarse spatial scale by weather surveillance radar (WSR) from a previous study, which were demonstrated to be strongly correlated at our study site [12]. We investigated patterns of nocturnal traffic rates measured at a series of time intervals throughout the night on preceding and following nights relative to daily counts to examine if traffic estimates closer to sunrise were more indicative of stopover density of the following morning, or whether early evening estimates were more strongly related to stopover density from the prior morning (sensu [10]). We predicted that early morning nocturnal traffic estimates (i.e., migrant influx) would better represent early morning stopover density, rather than following evening traffic estimates (i.e., migrant exodus).

## Methods

### Study site and data collection

We studied migration in Lewes, Delaware (38°46’58.53”N, 75° 9’53.41”W), adjacent to Breakwater Harbor (∼500 m to the NE), and approximately 1 km from the northwest-southeast running coastline of the Delaware Bay [12]. Using methods described in Horton et al. [12] we sampled migrants during peak land bird migration from April 1^st^ to May 31^st^ and September 1^st^ October 31^st^ in 2011 and 2012. We used weather surveillance radar and thermal imaging to quantify nocturnal passage rates, and daily transect surveys to assess diurnal stopover density. We collected nightly data between evening twilight and morning twilight (sun 6° below the horizon). To account for day-to-day changes in night length, we used tenths of the night (deciles) rather than absolute time relative to sunset or sunrise. Sampling effort varied due to poor weather conditions, and by technique because of technical errors in recording equipment. We included partial nights if more than half of the night was sampled. To limit weather-related detection biases, nights with no or minimal precipitation were included in our analyses. For additional information describing migrant flight speeds, heights, and detection biases across these sampling periods see Horton et al. [12].

### Radar traffic estimates

We downloaded 1 km x 1 km resolution WSR-88D National Mosiac 3D composite unfiltered reflectivity data centered over the study site from the National Severe Storms Laboratory’s National Mosaic and Multi-Sensor QPE (NMQ) interface. At this location (range 24.5 km at an azimuth 101.5°) the airspace is sampled at an elevation range of 1 to 588 m above ground level every ten minutes during clear-air mode and every five minutes during periods of precipitation [18]. To exclude scans containing precipitation and anomalous beam propagation [19, 20], we inspected reflectivity data using the Surveillance of the Aerosphere Using Weather Radar website (http://soar.ou.edu/legacy.html).

To identify bird- from insect-dominated nights, we determined mean target airspeed by vector-subtracting the wind velocity from the calculated target ground velocity. To estimate the mean ground speed of flying animals we first fit sine functions to annular rings of radial velocity measures at each range distance from the radar following [21]. We used level-II KDOX radial velocity data from a single 2.5° elevation angle sweep collected approximately three hours after local sunset that we downloaded from the National Centers for Environmental Information (NCEI). To determine winds aloft we obtained radiosonde data from Wallops Island, VA (~110 km from site) through the University of Wyoming, Laramie archive. We determined air speeds at heights corresponding to height measures of radiosonde, then computed mean air speed by weighting speeds at each height interval by the relative density of animals at each interval based on vertical profiles of reflectivity calculated following [22]. We considered radar scans with mean target airspeeds of greater than or equal to 4.5 m · s^−1^ as bird dominated [6, 23]. We used only bird dominated nights for analyses.

To derive a bird traffic rate from radar measures, we first converted the native radar reflectivity factor (Z) into reflectivity (cm^2^ · km^−3^) following [24]. Using an average passerine bird radar cross section of 15 cm^2^ for S-band radar [25], we converted reflectivity into a measure of volumetric passerine density aloft (birds · km^−3^). Finally, we took the product of volumetric bird density, mean ground speed of birds three hours after local sunset derived from radial velocity data, and the estimated cross-sectional area of the radar beam above the site (0.27 km^2^) to derive a Migration Traffic Rate (MTR) in terms of number of passerines crossing a 1-km line within an hour (birds · km^−1^ · hr^−1^).

### Thermal infrared imaging traffic estimates

We used a FLIR Guardsman HG-307 Pro thermal infrared camera with a 7° field of view and 320 x 240 pixel resolution. We mounted the camera in vertical orientation to detect overhead flight activities of all birds, bats, and arthropods. For this device small songbirds (i.e., 14 cm length; e.g., Yellow-rumped Warbler, *Setophaga coronata*) exhibit a maximum sampling range of ~375 m, which at this range occupy two pixels. For a larger sized songbird (i.e., 20 cm length; e.g., Wood Thrush, *Hylocichla mustelina*) the maximum detection range is estimated to be ~530 m. All video was manually screened (viewing speed ≤ three times real time) on a desktop PC to determine the numbers of individuals aloft and when possible taxa identification (bird, bat, or insect). All suspected bat and insect targets (e.g., irregular flight patterns) were removed for analysis.

We calculated traffic estimates as the number of targets passing the field of view per hour. We were unable to calculate a standard migration traffic rate because the maximum detection range varied nightly due to weather conditions and accurate flight elevation measurements could not be determined.

### Stopover density estimates from transect counts

We used a 500 m long by 50 m wide strip transect survey to estimate bird density in a shrubland habitat dominated by Atlantic white cedar (*Chamaecyparis thyoides*), southern wax myrtle (*Myrica cerifera*), and American holly (*Ilex opaca*) with a 6–10 m tall canopy. Observers conducted counts daily (weather permitting) during both spring and fall of 2011 and 2012. Observers were the same for spring and fall 2012, but differed for the other seasons. Additionally, two observers were used during spring 2011. Observers documented species, number of individuals, and perpendicular distance from the transect centerline of all bird observations classified into bins of 0–5 m, 5–10 m, or 10–25 m. The transect was walked in ∼ 30 min duration, and we alternated the direction in which the transect was walked on repeat surveys. We included only nocturnally-migrating birds in our bird stopover density estimates. To account for variable detection probability from transect counts across distance classes we determined detection probability and migrant density estimates within R using the “unmarked” package [26, 27].

### Mist-netting

We captured birds using passive mist-netting from September 1^st^ to October 26^th^, 2012. We operated 2 to 16 (6–12 × 2.6 m) 30-mm mesh mist nets within the 500 m strip transect area. Nets were opened at sunrise and closed six hours later with checks conducted every 30 min. We fit all birds with a U.S. Fish and Wildlife Service aluminum band and recorded mass within 0.01 g, wing chord within 0.5 mm, fat class scored from 0 = no fat to 5 = bulging over breast and abdomen, date and time of capture, and age and sex when possible. Mist-netting was dependent on suitable weather conditions and net closure prompted upon precipitation, high winds, dense fog, and extreme ambient temperature. Netting effort was also variable in order to limit capture rate because only one person operated the nets (i.e., high initial capture rate prompted complete or partial net closure). To correct for variable sampling effort we calculated birds · nethour^−1^ as an estimate of stopover intensity. Only newly banded nocturnally-migrating birds were included in stopover density estimates. Because our mist-netting efforts were limited to the fall of 2012, we only correlated banding measures with transect counts to ensure they yielded similar traffic indices.

### Statistical analyses

We examined two aspects of migrant passage 1.) daily magnitude and 2.) daily flux. We tested for correlations between the daily magnitude of traffic rates and stopover densities (via transect counts) that would elucidate the non-stationary seasonal trend in migration activity. To recast traffic rates and stopover densities as fluxes we removed the seasonal trend in the time series (i.e., made the time series stationary) by computing the differences between consecutive observations. We used the “zoo” package in R to calculate detrended time series [28]. We assessed these relationships (magnitude and flux) between methods on the nights preceding diurnal counts and the nights following these counts using Bayesian Pearson’s correlations on migratory traffic and stopover density estimates. We performed correlations using both nightly means of traffic estimates and means for decile periods of the night. We related diurnal stopover density estimates to migration traffic rates observed during the preceding night or the following night.

We implemented all analyses using Markov Chain Monte Carlo simulations (MCMC) using JAGS program for analysis of Bayesian correlations [29] via the “rjags” package [30]. Flat priors were used for each of the parameters because no prior expectation was appropriate for these analyses. To buffer against the influence of outliers, a multivariate t-distribution (dmt) was used within the model specification, a motivating factor for using these Bayesian analyses. Each individual correlation was run using two chains, with a burn-in of 500 samples and a total of 5000 samples monitored for posterior estimates of the correlation coefficient (r). Thinning was executed to maintain every 2^nd^ MCMC iteration sample to reduce serial autocorrelation among samples. Sample chains were examined to ensure thorough mixing of Markov chains and stationarity of the posterior distribution assessed using with the Gelman-Rubin diagnostic [31]. From the posterior distribution of the samples the mean and Bayesian credible intervals were calculated for each pairwise comparison.

Because fall diurnal indices were dominated by the presence of Yellow-rumped Warbler (*Setophaga coronate*), we examined their influence on trended (magnitude) and detrended (flux) daily correlation strength, both for the preceding night and following night. To quantify the impact we randomly subsampled each data set through 500 iterations, retaining 25 nights for radar comparisons and 15 for thermal imaging. For each iteration we monitored the correlation strength and mean daily proportion of Yellow-rumped Warblers on transect counts. We used a linear model to assess the influence of Yellow-rumped Warbler predominance on correlation strength.

## Results

### Nocturnal traffic estimates

Using weather surveillance radar, seasonal migration traffic rate averaged 1420 birds · km^−1^ · hr^−1^ · night^−1^ during the spring (range = 11–9558 birds · km^−1^ · hr^−1^, *n* = 74) and 2050 birds · km^−1^ · hr^−1^ · night^−1^ during the fall (range = 41–8116 birds · km^−1^ · hr^−1^, *n* = 47). The thermal imaging camera detected 22 detections · hour^−1^ · night^−1^ during the spring (range = 9–131 detections · hour^−1^, *n* = 70) and 69 detections · hour^−1^ · night^−1^ during the fall (range = 4–252 detections · hour^−1^, *n* = 47).

### Stopover density estimates

**Fig. 1:**
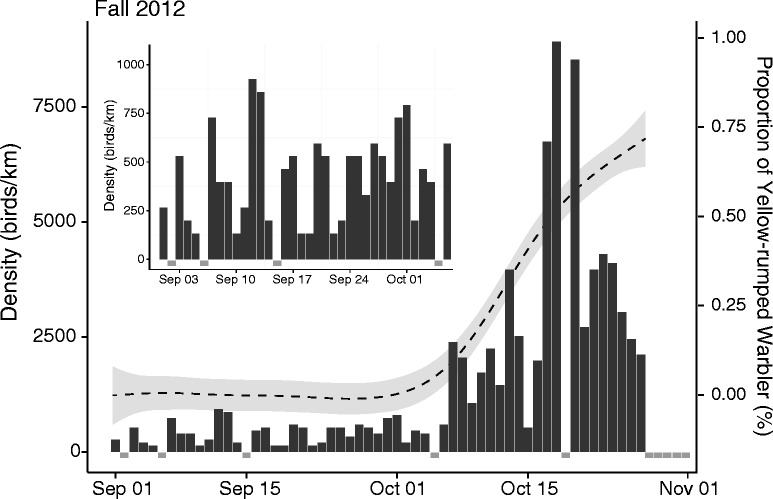
Fall diurnal migration phenology via transect counts. Fall 2012 mean daily transect count estimates of migrant density. Smoothed line represents the proportion of total birds detected as Yellow-rumped Warblers on daily counts. Inset figure displays migratory phenology from September 1^st^ to October 6^th^, 2012. Gray bars indicate missing data

We detected 1069 migrants during spring (mean = 9.9 migrants · day^−1^, *n* = 108) and 2564 during the fall (mean = 25.4 migrants · day^−1^, *n* = 101) during daily transect counts. Species composition varied by season, with 46 species detected during the spring and 65 species during the fall, although the top five most common species remained relatively consistent (Table 1). Yellow-rumped Warblers were the most abundant species observed, especially dominating late-season fall surveys (Fig. 1). Through mist-netting we captured a total of 2117 migrants of 48 species through 53 days of banding operation. We assessed both species richness and relative density measures between mist-netting and transect counts during the fall 2012 season. We found positive relationships between both metrics, although a much stronger correspondence with relative density (richness: *r* = 0.43, CI 0.43 to 0.65; density: *r* = 0.78, CI 0.62 to 0.89). Although we were unable to assess daily turnover using transect counts, our between-day recapture rates from our banding records were low (14.3 %), indicating that most migrants departed the site within one day.

**Table 1:** Top five species sampled on transect counts

Season	Common name (*scientific name*)	Number of individuals detected	Percent of total detected
Spring			
	White-throated Sparrow (*Zonotrichia albicollis*)	305	28.5
	Common Yellowthroat (*Geothlypis trichas*)	166	15.5
	Gray Catbird (*Dumetella carolinensis*)	156	14.6
	White-eyed Vireo (*Vireo griseus*)	79	7.4
	Yellow-rumped Warbler (*Setophaga coronata*)	66	6.2
Fall			
	Yellow-rumped Warbler (*Setophaga coronata*)	1228	47.9
	Gray Catbird (*Dumetella carolinensis*)	494	19.3
	Common Yellowthroat (*Geothlypis trichas*)	89	3.5
	Golden-crowned Kinglet (*Regulus satrapa*)	82	3.2
	White-throated Sparrow (*Zonotrichia albicollis*)	81	3.2

Raw counts of the five most common migrants detected on daily transect counts from spring (2011–12) and fall (2011–12) in Lewes, DE

### WSR traffic rate to stopover density comparisons

For nightly means, we found moderately-strong positive correlations between the magnitude of stopover density and radar MTR for all night and season combinations, whereas only a single moderately-strong correlation between flux of stopover density and radar MTR was found (Table 2). During the fall, we found that mean daily correlation strength for magnitude comparisons decreased by 0.0029 (±0.0019; ±95 % CI) on preceding nights and by 0.0072 (±0.0014; ±95 % CI) on following nights with increasing percentage of Yellow-rumped Warbler occurrence. We found no changes in daily comparisons of mean fluxes with increasing percentage of Yellow-rumped Warbler occurrence on preceding (−0.000949 ± 0.0028; mean ±95 % CI) or following nights (−0.0014 ± 0.0028; mean ±95 % CI).

**Table 2:** Spring and fall nightly correlations preceding and following daily transect counts

Season	Method	Metric	Preceding night			Following night		
			*r*	95 % CI	n	*r*	95 % CI	n
Spring	WSR	Magnitude	**0.316**	**0.021 to 0.572**	**65**	**0.435**	**0.145 to 0.672**	**61**
		Flux	0.161	−0.121 to 0.428	63	**0.547**	**0.324 to 0.726**	**59**
	TI	Magnitude	0.015	−0.238 to 0.265	68	0.174	−0.127 to 0.452	61
		Flux	0.059	−0.206 to 0.320	66	**0.598**	**0.386 to 0.760**	**59**
Fall	WSR	Magnitude	**0.538**	**0.292 to 0.734**	**47**	**0.457**	**0.186 to 0.677**	**47**
		Flux	−0.143	−0.442 to 0.172	45	−0.010	−0.334 to 0.315	45
	TI	Magnitude	0.067	−0.481 to 0.566	26	0.004	−0.481 o 0.481	26
		Flux	−0.001	−0.439 to 0.437	24	−0.029	−0.457 to 0.403	24

Bayesian Pearson’s correlation coefficients (r) of pairwise correlation tests among migration traffic rates across nights during the spring (2011–12) and fall (2011–12). Daily mean stopover estimates were correlated with mean nocturnal traffic estimates preceding the transect count and following to the transect count. Comparisons were made on trended (magnitude) and detrended (flux) traffic and stopover measures. Credible intervals not overlapping zero are highlighted in bold. WSR = weather surveillance radar, TI = thermal infrared camera

**Fig. 2:**
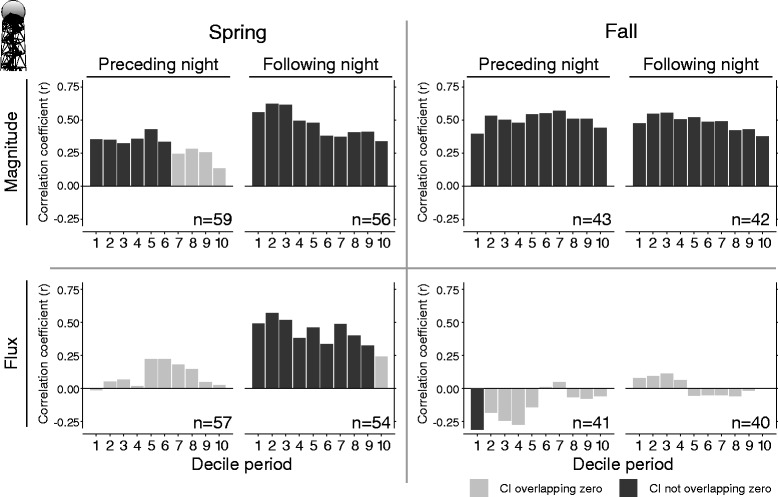
Decile comparisons of radar migration traffic rates preceding and following diurnal transect counts. Spring and fall Bayesian Pearson’s correlation coefficients (r) of pairwise correlation tests between decile measures of radar MTR preceding and following diurnal transect counts. Comparisons were made on trended (magnitude) and detrended (flux) traffic and stopover measures. Minimum sample sizes are labeled for each comparison. Credible intervals not overlapping zero are signified by black bars, and those overlapping zero denoted by gray bars

We conducted comparisons using decile periods of the night, which provided some nuance to the comparisons of nightly means (Fig. 2). Radar comparisons of magnitude in MTR tended to peak during the middle or later half of preceding nights (decile 5 and 7), but earlier in the night (decile 3 and 2) during following nights in spring and fall, respectively. The strength of correlations of magnitude in MTR were rather consistent between preceding and following nights in fall, but stronger during following nights in spring. Positive correlations of fluxes were strongest during decile 2 of spring following nights. The only credible correlation between fluxes during fall occurred during the first decile of preceding nights and was negative.

### TI traffic rate to stopover density comparisons

**Fig. 3:**
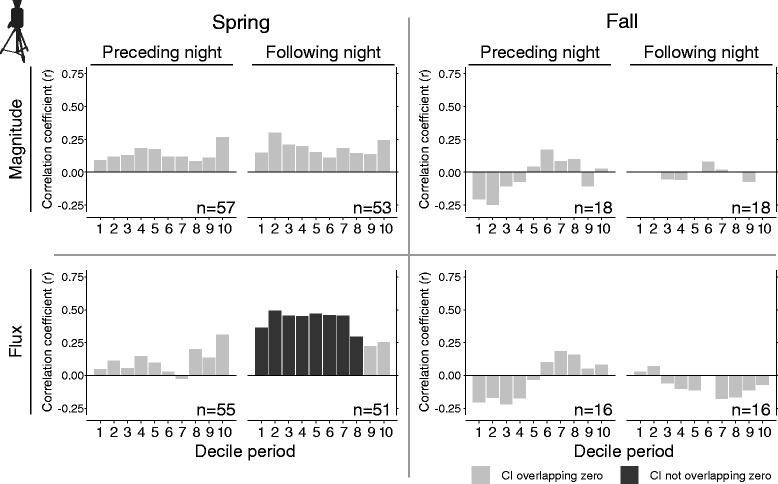
Decile comparisons of thermal imaging migration traffic rates preceding and following diurnal transect counts. Spring and fall Bayesian Pearson’s correlation coefficients (r) of pairwise correlation tests between decile measures of thermal imaging traffic estimates preceding and following diurnal transect counts. Comparisons were made on trended (magnitude) and detrended (flux) traffic and stopover measures. Minimum sample sizes are labeled for each comparison. Credible intervals not overlapping zero are signified by black bars, and those overlapping zero denoted by gray bars

Unlike WSR, TI comparisons of nightly means yielded no correlations that were credibly different from zero between magnitude of stopover density and TI MTR for any night and season combinations (Table 2). However, a moderately-strong correlation between flux of stopover density and TI MTR occurred during spring on following nights, which was similar to WSR. Follow-up analysis of TI MTR and stopover density comparisons between decile periods of the night during spring revealed that the positive correlations with fluxes on following nights was greatest during the 2^nd^ decile of the night and remained consistently high until the 8^th^ decile (Fig. 3). There were no credible correlations during deciles periods of the night for any fall comparisons.

## Discussion

Diurnal bird counts during stopover were positively related to broad-scale traffic rates of migrants on preceding and following nights during the spring and fall. The relationships between these metrics are likely driven by the overarching seasonal phenology in migration activity. However, removing the broad temporal trend in migration phenology (i.e., comparing daily fluxes) revealed fine-scale differences in how these metrics relate between spring and fall, which may indicate how migrants differentially use the study site between seasons.

The spatial scale of nocturnal traffic measures relative to the extent of the area surveyed for stopover incidence was important. The moderate correlation of stopover incidence with broad-scale WSR traffic rates in the one square kilometer airspace over our site (1/40^th^ of the spatial extent of the radar measure), stands in stark contrast to the lack of correlation between stopover incidence and the number of animals passing overhead in the narrow-beam TI camera. The surprisingly weak relationship between TI traffic rate and stopover density is also counter to Zehnder and Karlsson [13] who found relatively strong correlations between bird banding counts and TI detections. Because TI represents a fine-scale site-specific traffic estimate, we predicted it would facilitate the strongest correlations. However, thermal imaging can have severe atmospheric biases, and low precision caused by low detection rates within the narrow sampling beam -- both of which can diminish its accuracy to capture localized traffic rates [12].

### Seasonal trend in migration phenology

The collective seasonal trends in migration phenology of stopover density for all migrants combined was most consistently and strongly correlated with peak migration traffic rate on following nights among seasons. This is counter to our prediction that correlation strength between traffic estimates on preceding nights and diurnal stopover densities the following day would increase during the night, peaking just before the last birds aloft made landfall as Peckford and Taylor [10] had previously found. The weaker preceding night relationships may be explained because we are unable to quantify the proportion of passing nocturnal migrants making landfall at our site, and because daily stopover density is likely cumulative, traffic estimates preceding stopover may not be entirely indicative of stopover density. However, because many migrants initiate departure flights from daytime stopover sites *en masse* near evening civil twilight [32], early measures of traffic rates (i.e. deciles 1 and 2) may be more indicative of cumulative migrant stopover density, and thus yield stronger comparisons when using metrics from the following night.

### Migration flux within season

Spring migratory movements tend to show a rapid phenology, typified by protracted stopover times [33] and increased flight speed relative to fall movements [34, 35]. Consistently favorable springtime winds [34], and strong selection pressure to arrive early on the breeding grounds [36] facilitate contracted seasonal movements. Thus, it is this not surprising that when examining daily flux, an indicator of turnover, we found strong seasonal dependence on pairwise comparisons. In the spring, broad (WSR) and fine-scale (TI) measures of flux in traffic rate on following nights closely (correlation coefficient: 0.547–0.598) matched the flux in densities on the ground. This relationship could arise if the proportion of birds leaving the site (i.e., turnover rate) was consistent from night-to-night throughout the season. The lack of correlation on preceding nights suggests that the local influx of birds on the ground is not related to fine- or broad-scale numbers flying over during the night. This may indicate that our site is not an attractive spring stopover site (i.e., poor habitat quality) and/or landfall is shaped by other factors (e.g., weather) during the night. For example, landbird migrant stopover density in the region during fall is strongly correlated with the amount of forested habitat [37]. Despite detecting many forest-dwelling migrants in the shrub habitat that we sampled, migrants may have preferred to land in forest habitats nearby.

In contrast to spring, the lack of positive fall correlations with flux on following nights may be related to inconsistent turnover of migrants at the site relative to spring [35]. Furthermore, high traffic rates of birds early during preceding nights in fall were correlated with a large decrease in the numbers of migrants stopping over. Thus, large local departures of birds from the site were followed by low migrant counts (i.e., emptying of the site). During the fall some migrants appear to wait for favorable winds, resulting in more episodic migration activity and a build-up of individuals at sites over multiple days [35, 38–41], consistent with the flux patterns we observed.

### Complexity of comparing traffic rates and stopover incidence

Any comparison of nocturnal traffic rates and diurnal stopover incidence of migrating landbirds is complex. These parameters represent fundamentally different aspects of migration activity, making the leap from magnitude or fluxes of migrants passing overhead to magnitude or fluxes of migrants on the ground dependent on many assumptions about bird behavior and how they are measured. Relationships among migrant bird flux measures may be more complex than simple measures of seasonal phenology of migration. For example, it is assumed that passage rate is related to the number of birds eventually making landfall in a given location. However, birds can be expected to make landfall at any point of the night, possibly due to adverse weather conditions [42–45], energetic condition [46], or other unforeseen endogenous and/or exogenous factors. Factors influencing the propensity of birds to land in a particular location, like poor weather, may be idiosyncratic and undermine this assumption. This can be manifested as noise when assessing seasonal trends of migration phenology (e.g., [13, 14]). Whether it completely confounds assessment of finer-scale flux in migration activity remains to be determined.

Migration strategies vary seasonally. Due to seasonally dependent turnover rates [33], the relationship between daily fluxes in migrant density with traffic rates is also seasonally dependent. The use of mark-recapture methods (i.e., bird-banding) can serve to improve the estimate of new arrivals (i.e., turnover). Continuous banding, especially during the breeding and wintering periods, would allow for the determination of true migrants. Some individuals of particular species, such as the Gray Catbird (*Dumetella carolinensis*), were known to breed at our site, while many others pass through *en route* to northerly breeding (spring) and southerly wintering (fall) grounds [47]. During our fall 2012 banding season we captured a total of 127 individuals of this species, yet the proportion of these individuals that could be classified as true migrants could not be assessed using our banding methodology. Even with these concerns we did find strong congruence between fall 2012 transect and mist-netting density estimates.

Other factors can influence nocturnal traffic rates that can weaken the correlation with diurnal stopover incidence. For one, we do not have certainty of taxonomic (i.e., bird v. insect), and especially species composition of flying animals on a given night [48, 49]. We attempted to minimize contamination from non-avian (i.e., insect) targets using flight speeds derived from weather surveillance radar to screen the dominate flying taxa [23, 50], and when possible removed individual insect and bat detections from the thermal imaging record. However, non-avian targets were not completely removed from the data. Further exploration and advancement of taxonomic classification criteria (e.g., airspeed) is vital for future studies in this arena. Additionally, thermal infrared cameras have variable detection ranges depending on bird size, a factor that could introduce more noise in TI traffic rates as compared to WSR estimates. This may help explain the weaker correlations with TI and ground data.

Lastly, study site placement and lack of spatial replication undoubtedly limits the generality of our findings. The dominant vegetation at the study site (i.e., *Myrica cerifera* and *Chamaecyparis thyoides*), as well as geographic location, were likely responsible for the large proportion of Yellow-rumped Warblers (*Setophaga coronata*) detected during our fall sampling seasons [47]. We observed a great influx of this species in late fall (85.6 % of detections collected between October 13^th^ to October 26^th^), comprising 47.9 % of fall migrant detections, 35.6 % of all migrant detections. We believe most were migrants as few (4.8 %) were recaptured during our fall 2012 banding effort. Curiously the passage of this species was not obvious from nocturnal traffic metrics. Yellow-rumped Warblers tended to weaken correlation strength for preceding and following night comparisons, however not consistently for each method and metric. Study site habitat likely worked to attract this particular species, while possibly excluding others. Furthermore, by only sampling stopover incidence within one habitat type, we only detected a subset of all bird species migrating overhead. For example, both waterfowl and shorebirds were not detected on transect counts, although both were likely detected by radar and thermal camera. However, we focused our sampling during the peak of landbird migration to limit the contribution of other bird taxa to traffic rate measures. To the extent that the phenology in stopover incidence of the bird species we detected was representative of the entire migrant bird community during the sampling period is uncertain, but it was sufficient to provide moderate correlations with migrant traffic rates aloft. Future sampling of stopover incidence across multiple habitat types may provide a more complete assessment of the pool of migrant species moving through a region and provide for tighter correlations between ground and air measures.

## Conclusions

Multi-year diurnal stopover sampling methods have been suggested to be used as indicators of migrant population trends [1, 2, 51]. Thus, it is important to know how the daily density of migrants stopping over relates to the number of migrants passing overhead on a given night. Numerous factors can influence both traffic and stopover estimates in complex ways that are beyond the control of researchers. We have shown that the order of these comparisons (preceding or following day), traffic metric (magnitude or flux), and timing of these comparisons through the night can have important consequences on correlation strength and direction. Additionally, we showed that monitoring techniques (WSR or TI) yield varying depictions of migratory activity, with broad-scale weather surveillance radar measures yielding the strongest pairwise comparisons at our site. Given the complexity of these comparisons, we advocate continued investigation using multiple sites spanning a diversity of habitats, and geographic localities (e.g., inland, coastal) to improve our understanding of how best to monitor migration phenology and flux.

### Availability of supporting data

The data sets supporting the results of this article are included within the article and its additional files.
